# Neonatal Metformin Exposure Is Associated with Altered Adult Metabolic Responses to Hyperlipidic Diet in Male Wistar Rats

**DOI:** 10.3390/life16091398

**Published:** 2026-08-24

**Authors:** Gustavo Henrique Doná Rodrigues Almeida, Claudio Guilherme de Assis Oliveira, Bianca Fuzeti Candian, Scarlett Rodrigues Raposo, Leticia Ferreira Barbosa, João Victor Damin, Beatriz Marques Ferreira, Lucas Paulo Jacinto Saavedra, Douglas Lopes de Almeida, Leandro Martins de Freitas, Silvano Piovan, Paulo Cézar de Freitas Mathias

**Affiliations:** 1 Experimental Laboratory in DOHaD (LEx DOHaD), Graduate Program in Biological Sciences, State University of Maringá, Maringá 87020-900, PR, Brazil; ra136991@uem.br (C.G.d.A.O.); ra135763@uem.br (B.F.C.); scarlettrraposo@gmail.com (S.R.R.); pg55995@uem.br (L.F.B.); pg405653@uem.br (J.V.D.); ra134899@uem.br (B.M.F.); dlalmeida@uem.br (D.L.d.A.); silvano.piovan@univel.br (S.P.); 2 Laboratory of Development and Innovation, Butantan Institute, São Paulo 05503-900, Brazil; 3 Weill Cornell Medicine, Cornell University, Ithaca, NY 14853, USA; 4 Department of Biochemistry, State University of Maringá, Maringá 87020-900, PR, Brazil; 5 Multidisciplinary Institute of Health, Federal University of Bahia, Vitória da Conquista 45029-094, BA, Brazil; leandromartinsdefreitas@gmail.com; 6 Department of Biochemistry, State University of West Paraná, Cascavel 85819-110, PR, Brazil

**Keywords:** DOHaD concept, obesity, metformin, metabolic programming

## Abstract

Obesity is a multifactorial disorder with increasing global prevalence, highlighting the importance of identifying early-life factors that may influence long-term metabolic outcomes. While metformin is widely used to improve metabolic dysfunction in adulthood, whether neonatal exposure to this drug modifies adult responses to subsequent dietary challenges remains poorly understood. This exploratory study evaluated whether intraperitoneal metformin administration during the neonatal period alters adult metabolic responses to hyperlipidic diet (HL) exposure in Wistar rats. Following weaning and sex determination, male offspring (*n* = 80) were included in the study and distributed among four experimental groups, i.e., saline–normolipidic (SAL-NL), saline–high-fat (SAL-HL), metformin–normolipidic (MET-NL), and metformin–high-fat (MET-HL), with 20 animals per group. From postnatal days 1 to 12, animals received daily intraperitoneal saline or metformin (100 mg/kg/day). From days 60 to 90, animals were fed normolipidic or hyperlipidic diets. Body mass, food intake, glucose tolerance (GTT), and insulin tolerance (ITT) were assessed. Neonatal metformin exposure was associated with reduced body mass gain in MET-HL animals compared to SAL-HL, while food intake differed according to dietary condition. Metformin-treated animals also exhibited improved insulin sensitivity, reflected by higher kITT values, while no significant differences were observed in glucose tolerance. These preliminary findings suggest that neonatal metformin administration may influence adult metabolic responses to dietary challenge. Further studies are required to elucidate the mechanisms and long-term implications of this early-life intervention.

## 1. Introduction

Early-life environmental exposures play a fundamental role in shaping long-term metabolic health, as proposed by the Developmental Origins of Health and Disease (DOHaD) concept. According to this framework, nutritional, hormonal, and environmental stimuli acting during critical developmental windows induce adaptive responses that may persist throughout life and influence susceptibility to metabolic disorders, including insulin resistance, type 2 diabetes, and obesity [1,2,3,4]. Consequently, identifying interventions capable of modulating these early-life adaptive processes has become an important focus of experimental metabolic research.

Obesity remains one of the most prevalent chronic metabolic disorders worldwide and represents an important experimental model for investigating developmental metabolic programming [4]. In addition to genetic predisposition, environmental exposures during critical periods of development substantially contribute to obesity susceptibility by altering energy balance regulation, insulin sensitivity, adipose tissue expansion, and neuroendocrine pathways [5,6,7].

Among these developmental stages, lactation represents a particularly critical window, especially in rodents, during which there is intense neuronal proliferation and differentiation, maturation of the hypothalamic–pituitary axis, establishment of regulatory circuits for energy metabolism, and rapid expansion of adipose tissue [8,9]. During this period, adverse environmental stimuli such as excessive nutrient supply, maternal stress, or exposure to xenobiotics can exert profound and lasting effects on the developing organism. These exposures may interfere with the maturation of neuroendocrine pathways that regulate appetite and energy expenditure, alter adipose tissue expansion and remodeling, and impair insulin and lipid signaling in peripheral organs [10,11].

These changes occur, in part, through epigenetic mechanisms such as DNA methylation, histone modifications, and regulation by microRNAs, which modulate gene expression without altering the DNA sequence [12,13,14]. Evidence suggests that early-life nutritional and hormonal exposures may influence epigenetic regulation in key metabolic tissues, including the liver, brain, muscle, and adipose tissue, thereby contributing to long-term metabolic outcomes [15].

In addition to dietary influences, pharmacological interventions during critical stages of development have emerged as promising tools for investigating developmental metabolic programming [16]. Among pharmacological agents capable of influencing metabolic homeostasis, metformin has attracted increasing attention beyond its conventional use for type 2 diabetes [17]. In addition to improving insulin sensitivity and reducing hepatic glucose production, experimental evidence suggests that metformin may modulate cellular pathways involved in metabolic adaptation, including AMPK activation, mitochondrial function, and inflammatory responses [18,19]. These properties have stimulated interest in its potential application during critical developmental periods.

Despite increasing interest in the use of metformin beyond glycemic control, its potential as an early-life pharmacological intervention remains incompletely understood. Most experimental studies have evaluated metformin as a therapeutic strategy after metabolic dysfunction has already been established or in specific models of neonatal overfeeding [20]. Whether transient neonatal exposure to metformin, independent of an early nutritional insult, is sufficient to modify adult metabolic responses to a later obesogenic challenge remains unclear.

In this context, preclinical studies have demonstrated beneficial metabolic effects of metformin when administered during early developmental stages [21,22,23]. Supporting this rationale, our group previously demonstrated that neonatal intraperitoneal metformin administration during the first 12 postnatal days improved body weight regulation, adiposity, insulin sensitivity, and food intake in male rats subjected to neonatal overfeeding through the small-litter model [24]. However, because the small-litter model is itself a well-established model of developmental metabolic programming induced by early nutritional excess, it remains unclear whether neonatal metformin exposure alone is sufficient to induce persistent metabolic adaptations capable of modifying adult responses to a subsequent hyperlipidic diet challenge. We hypothesized that transient neonatal metformin exposure could modify adult metabolic responses to a subsequent hyperlipidic diet challenge. Thus, neonatal metformin administration was investigated here as an experimental pharmacological exposure to probe developmental metabolic plasticity, rather than as a proposed preventive intervention.

## 2. Materials and Methods

### 2.1. Experimental Design

All animal procedures were approved by the Committee on Ethical Conduct in the Use of Animals in Experimentation (CEAE) of the State University of Maringá (protocol no. 5869020418). Wistar rats were obtained from the Central Animal Facility of the university and maintained in the Sector Animal Facility of the Experimental Laboratory in DOHaD (LEx DOHaD) of the Department of Biotechnology, Genetics, and Cell Biology. Animals were housed in appropriate cages with free access to water and commercial chow, under controlled temperature (22 ± 2 °C) and a 12 h light/dark photoperiod. After a five-day acclimatization period, they were placed for mating at a ratio of three females per male. Pregnant females were subsequently housed individually until delivery. Animal experiments were designed, conducted, and reported in accordance with the ARRIVE 2.0 guidelines. The completed ARRIVE checklist is provided as Supplementary Materials.

**Figure 1:**
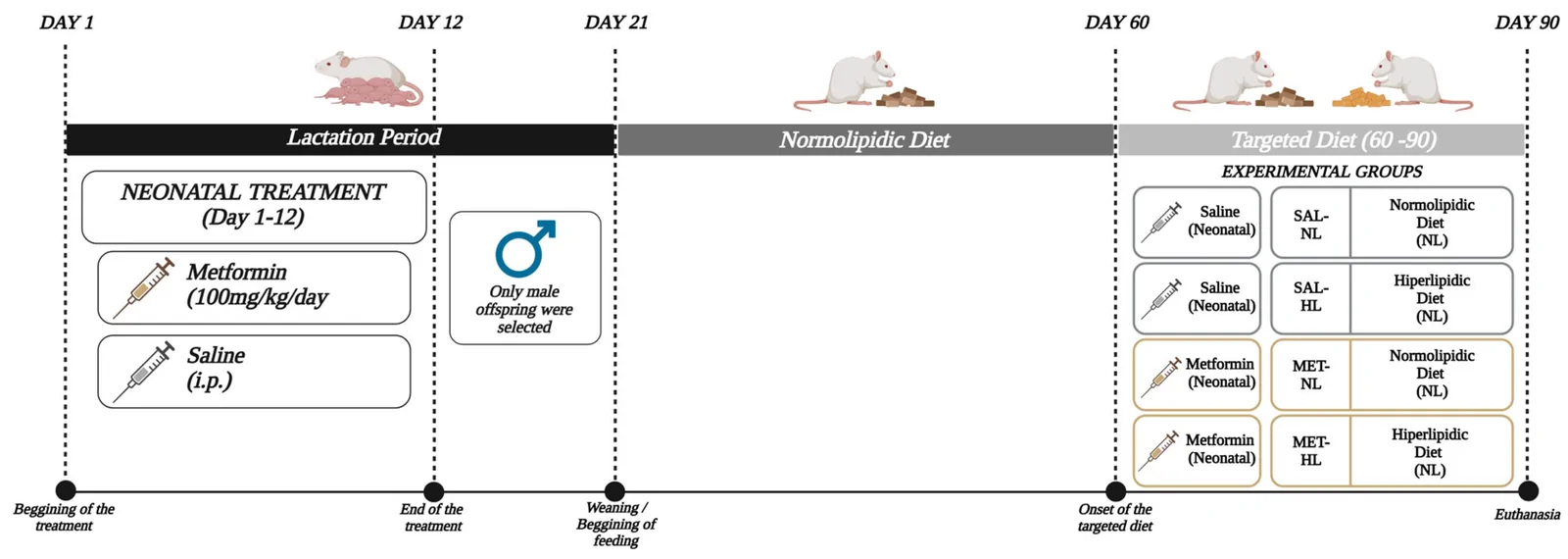
Experimental design of the study (Created in BioRender. Almeida, G. H. D. (2026) https://BioRender.com/puvz21k).

At birth, offspring were randomly assigned to receive either saline or metformin as the neonatal treatment. From postnatal day 1 to day 12, offspring assigned to the saline treatment received daily intraperitoneal injections of 0.9% NaCl, whereas those assigned to the metformin treatment received metformin (1,1-dimethylbiguanide hydrochloride, Sigma-Aldrich^®^, St. Louis, MO, USA) at a dose of 100 mg/kg body weight/day. The metformin dose of 100 mg/kg/day was selected based on previous experimental studies of early-life metformin exposure, including previous work from our group using this preclinical paradigm [24]. At 21 days of age, the offspring were weaned and sexed, and only male offspring were retained for the subsequent experimental protocol. A total of 80 male rats were included in the study and distributed among four experimental groups: saline–normolipidic diet (SAL-NL; *n* = 20), metformin–normolipidic diet (MET-NL; *n* = 20), saline–hyperlipidic diet (SAL-HL; *n* = 20), and metformin–hyperlipidic diet (MET-HL; *n* = 20). Animals were housed five per cage. Between 21 and 60 days of age, all animals received a standard normolipidic diet (Nuvital^®^, Curitiba, PR, Brazil). From day 60 to day 90, the SAL-HL and MET-HL groups were switched to a hyperlipidic diet (Table 1), whereas the SAL-NL and MET-NL groups remained on the normolipidic diet. The 4-week hyperlipidic dietary challenge was selected because previous studies have demonstrated that this exposure period is sufficient to induce early metabolic alterations in rats, including increased body weight, impaired insulin responsiveness, and disturbances in glucose homeostasis. The objective of this dietary intervention was therefore to provide a metabolic challenge rather than to establish a model of advanced or long-standing obesity [25]. Only male offspring were included to minimize the potential confounding influence of sex-specific hormonal fluctuations on metabolic outcomes and to maintain consistency with our previous studies using this experimental model. Future studies should evaluate whether female offspring exhibit similar responses to neonatal metformin exposure. The individual offspring was considered the experimental unit for the metabolic and physiological outcomes evaluated in this study. Litter was not included as an independent factor in the statistical analyses. Experimental analyses were conducted at 90 days of age. The overall experimental design is summarized in Figure 1.

### 2.2. Biometric Parameter Analysis

The body weight and food intake of all experimental groups were monitored throughout the study. At the end of the experimental period, animals were euthanized by decapitation using a sharp guillotine (Insight, Ribeirão Preto^®^, Ribeirão Preto, São Paulo, Brazil) to obtain whole blood samples for subsequent assessment of glycemic and insulin profiles. In addition, adipose tissue depots from the retroperitoneal, periepididymal, and mesenteric regions were excised and weighed. The choice of guillotine as the euthanasia method is supported by previous studies demonstrating that barbiturates can interfere with blood glucose levels, insulinemia, pancreatic islet function, and adrenal activity [26].

### 2.3. Fasting Blood Glucose Measurement

Basal plasma glucose levels were determined from samples collected after euthanasia by decapitation following a 12-h fasting period (*n* = 20 per group). Blood glucose was quantified using the glucose oxidase method by spectrophotometry with a semi-automatic biochemical analyzer (BIO200FL, Bio Plus^®^, São Paulo, SP, Brazil) and a commercial kit (Gold Analisa^®^, Belo Horizonte, MG, Brazil).

### 2.4. Intravenous Glucose Tolerance Test (ivGTT)

At 85 days of age, the animals were weighed and anesthetized with an intramuscular injection of ketamine/xylazine (75 mg + 15 mg/kg body weight). Subsequently, a silicone cannula was surgically implanted into the right external jugular vein. Through an anterior cervical incision, tissues were carefully dissected until the vein was exposed, and the cannula was introduced with the aid of an adapted needle. The cannula was secured to the pectoralis major muscle with a simple cotton suture and filled with a 10% heparin solution (Liquemine^®^, Basel, Switzerland, diluted in 0.9% NaCl) to prevent blood entry and clot formation. Following surgery, animals were housed individually in the animal facility and fasted for 12 h prior to undergoing the intravenous glucose tolerance test (ivGTT), performed 24 h post-surgery. At time zero, a baseline blood sample (400 µL) was collected via the cannula. Animals then received a glucose solution (1 g/kg body weight) administered through the cannula, after which additional blood samples (400 µL) were collected at 5, 15, 30, and 45 min. Plasma glucose concentrations were determined by the enzymatic-colorimetric method (GoldAnalisa^®^, Belo Horizonte, Brazil). The color reagent was added to plasma samples, incubated for 10 min at 37 °C in a water bath, and measured by spectrophotometry using a semi-automatic biochemical analyzer (BIO200FL, Bio Plus^®^, São Paulo, SP, Brazil). The ivGTT was selected to provide a standardized glucose challenge directly into the systemic circulation, minimizing variability associated with gastrointestinal glucose absorption and excluding the contribution of the incretin response. Although the OGTT more closely reflects physiological postprandial glucose handling, the ivGTT provides a controlled approach for assessing systemic glucose disposal and has been established for metabolic assessment in Wistar rats [27].

### 2.5. Intraperitoneal Insulin Tolerance Test (ITT)

After a 6-h fasting period, 87-day-old animals received an intraperitoneal injection of insulin (1 U/kg body weight). Blood samples were collected from the caudal vein, and glucose levels were measured using a glucometer (ACCU-CHEK^®^ Advantage, Roche Diagnostics, Mannheim, Germany) at 0 (baseline), 15, 30, 45, and 60 min after insulin administration. The blood glucose decay rate constant (kITT) was then calculated using the formula 0.693/(t^1^/_2_). The t^1^/_2_ of blood glucose was determined from the slope of the linear regression line of plasma glucose concentrations during the decline phase [28].

### 2.6. Statistical Analysis

The experimental group size (*n* = 20 animals per group) was established based on previous experience with this experimental model and on previously published studies from our group employing early-life metformin exposure and subsequent assessment of metabolic outcomes in male rats [24]. Given the exploratory nature of the present study, no single primary outcome was prespecified for a formal power-based sample-size calculation. The results are expressed as mean ± standard error of the mean (SEM). Data distribution was assessed using the D’Agostino–Pearson normality test prior to parametric statistical analysis, and the datasets analyzed using parametric tests satisfied the normality assumption. No data points were excluded as outliers, and no missing data were present in the datasets included in the statistical analyses. For longitudinal body weight measurements, the area under the curve (AUC) was calculated for each animal over the corresponding experimental period, thereby providing a single summary measure for statistical comparison. AUC values were compared between saline- and metformin-treated groups using an unpaired Student’s *t*-test. For the remaining comparisons, Student’s *t*-test or two-way analysis of variance (ANOVA) followed by Bonferroni’s post hoc test was used, as appropriate. Statistical analyses were conducted using GraphPad Prism software (version 7.0 for Windows; GraphPad Software, San Diego, CA, USA), and differences were considered statistically significant at *p* < 0.05.

## 3. Results

### 3.1. Body Weight and Food Consumption

**Figure 2:**
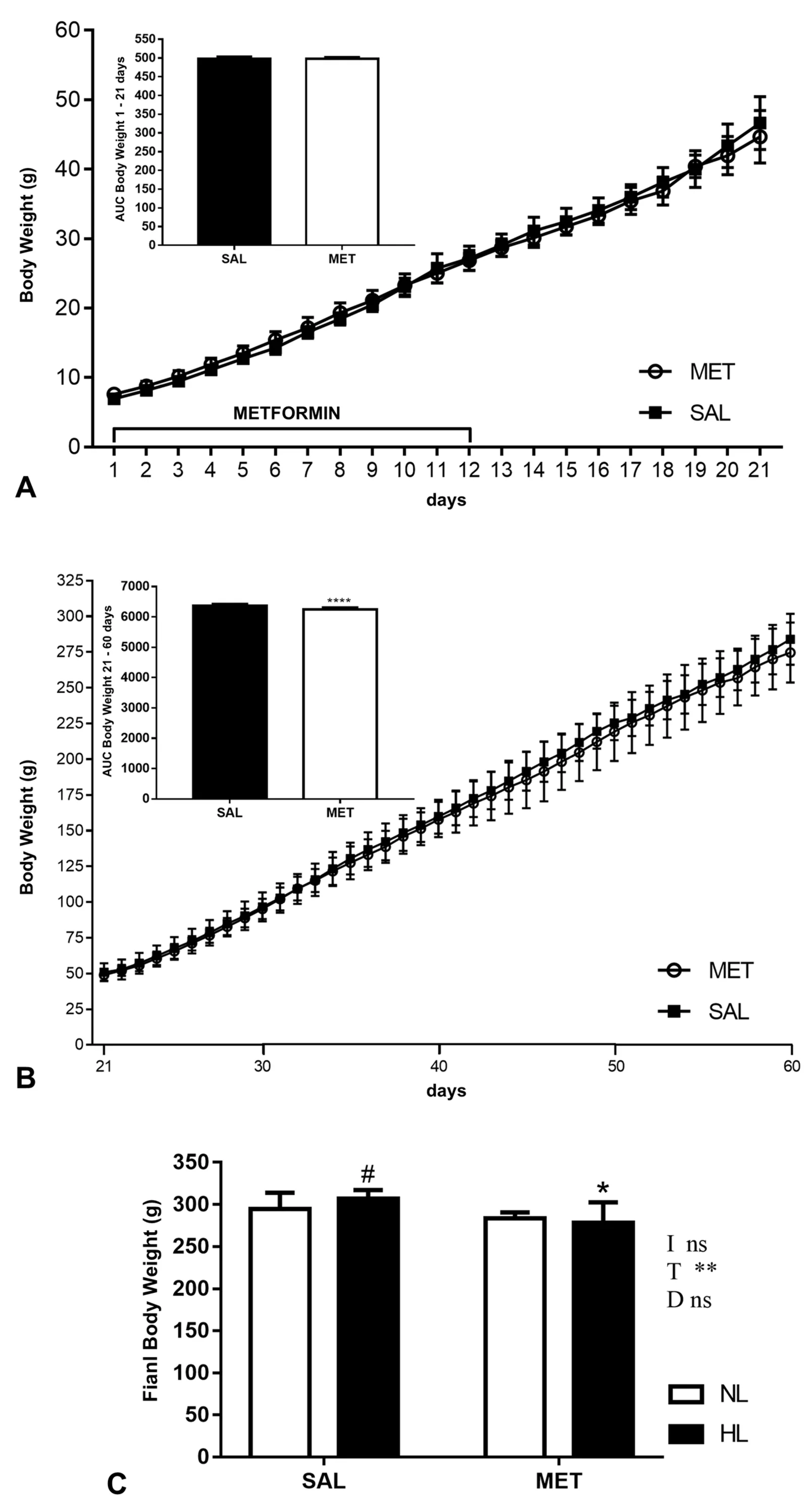
Body mass evolution of the experimental groups. (**A**) Body mass from day 1 to day 21 in animals treated intraperitoneally with saline (SAL) or metformin (MET). (**B**) Body mass from day 21 to day 60 in the same groups. (**C**) Final body mass at 90 days in SAL and MET groups fed normolipidic (NL) or hyperlipidic (HL) diets. Data are presented as mean ± SEM (*n* = 20/group). AUC, area under the curve. Statistical analysis: unpaired Student’s *t*-test (**A**,**B**) and two-way ANOVA followed by Bonferroni’s post hoc test (**C**). In panels (**A**,**B**), asterisks indicate significant differences between SAL and MET groups. In panel (**C**), # indicates a significant difference compared with the corresponding NL group, and * indicates a significant difference compared with the corresponding SAL group within the same dietary condition. I, treatment × diet interaction; T, treatment effect; D, diet effect; ns, not significant. # *p* < 0.05; * *p* < 0.01; ** *p* < 0.001; **** indicates *p* < 0.0001.

**Figure 3:**
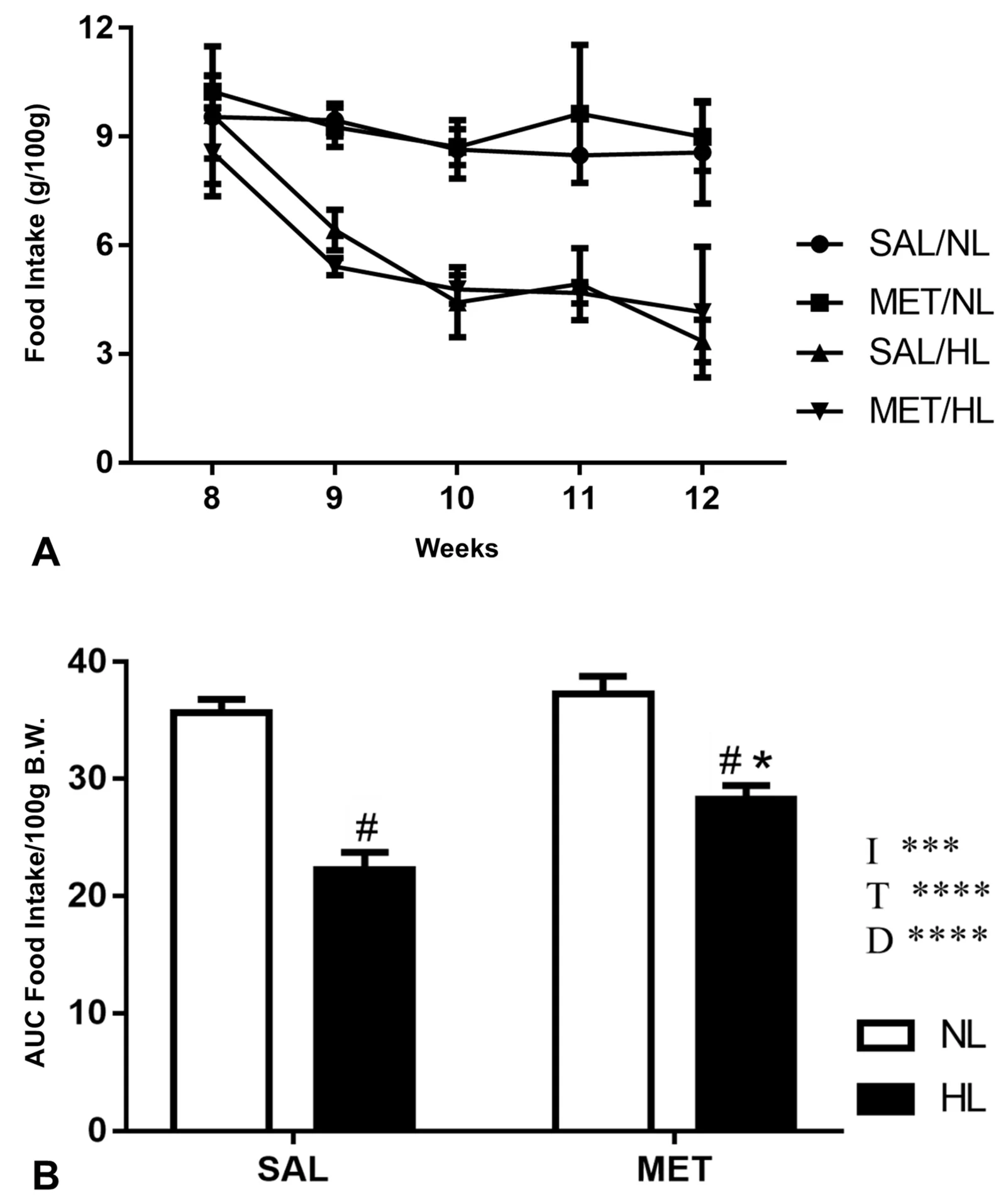
Evaluation of food intake in the experimental groups. (**A**) Food intake during the experimental period. (**B**) Area under the curve (AUC) of cumulative food intake. Data are presented as mean ± SEM (*n* = 20/group). Statistical analysis was performed using two-way ANOVA followed by Bonferroni’s post hoc test. # indicates a significant difference compared with the corresponding NL group, and * indicates a significant difference compared with the corresponding SAL group within the same dietary condition. I, treatment × diet interaction; T, treatment effect; D, diet effect; ns, not significant. # * indicates *p* < 0.05; *** indicates *p* < 0.001; **** indicates *p* < 0.0001.

From birth to day 21, corresponding to the period of intraperitoneal treatment with saline or metformin, no statistically significant differences were observed in the area under the curve (AUC) for body weight between the SAL and MET groups (Figure 2A). Between days 21 and 60, the MET group showed a lower body weight AUC compared to the SAL group (Figure 2B). At the end of the experimental period (90 days), animals in the SAL-HL group presented higher body mass than those in the SAL-NL group (Figure 2C). Among metformin-treated groups, MET-HL animals exhibited lower body mass compared to MET-NL; however, no statistically significant differences were detected between MET-HL and SAL-HL groups. Regarding food intake, the SAL-HL group displayed a lower AUC compared with the SAL-NL and MET-NL groups. The MET-HL group also showed a reduced AUC compared to both NL groups, and lower consumption than the SAL-HL group. No significant differences were found between MET-NL and SAL-NL animals (Figure 3).

### 3.2. Adipose Tissue Depot Weights

**Figure 4:**
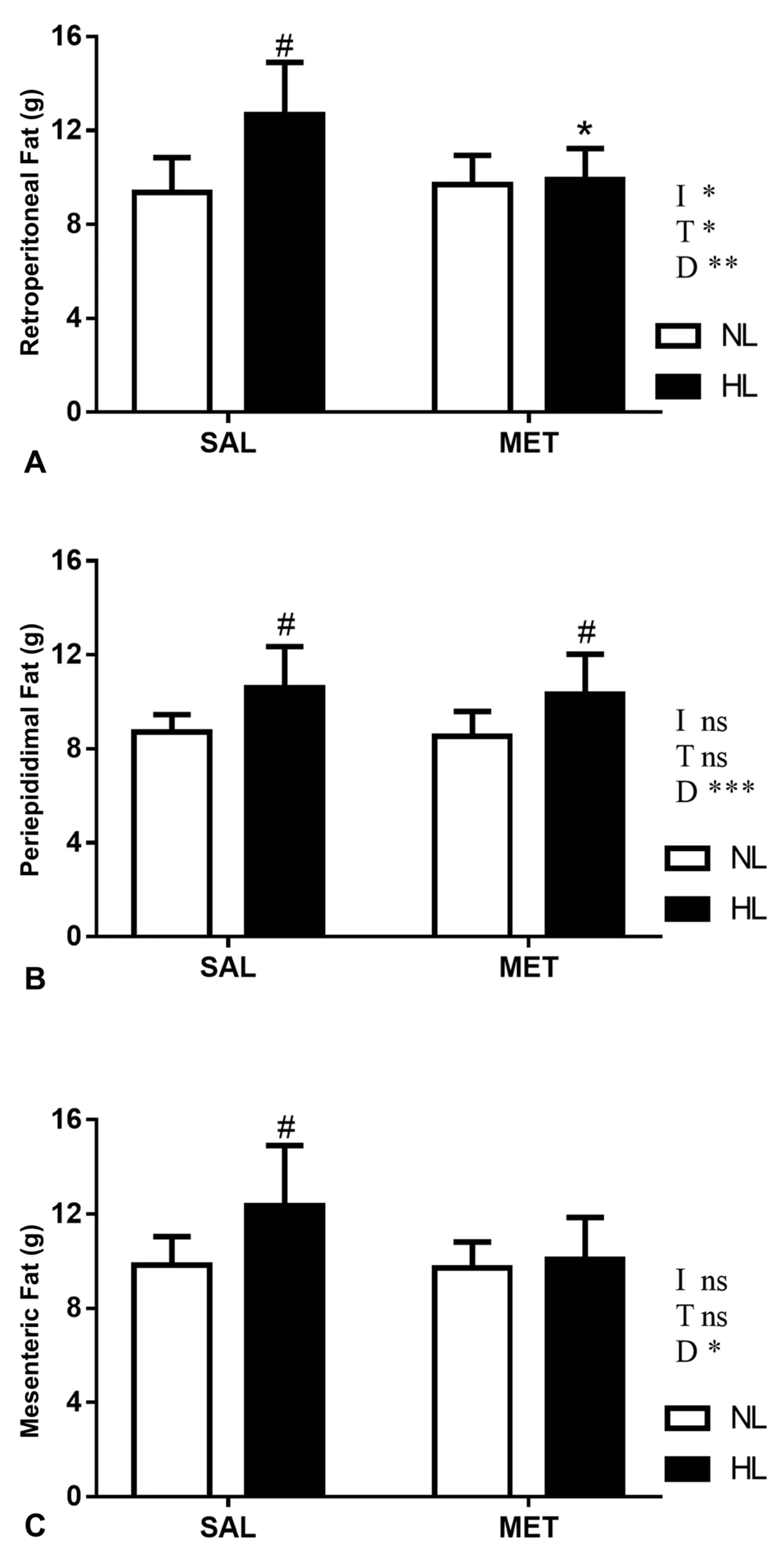
Quantification of retroperitoneal (**A**), periepididymal (**B**), and mesenteric (**C**) fat depot weights in the experimental groups. Data are expressed as mean ± SEM (*n* = 20/group). Statistical analysis was performed using two-way ANOVA to assess the effects of neonatal treatment and diet, followed by Bonferroni’s post hoc test. # indicates a significant difference compared with the corresponding NL group, and * indicates a significant difference compared with the corresponding SAL group within the same dietary condition. I, treatment × diet interaction; T, treatment effect; D, diet effect; ns, not significant. # *p* < 0.05; * *p* < 0.01; ** *p* < 0.001; *** *p* < 0.0001.

Figure 4 illustrates the effects of diet and neonatal metformin treatment on regional adipose tissue depot weights. The SAL-HL group exhibited higher retroperitoneal, periepididymal, and mesenteric fat weights compared to the SAL-NL group. Within the hyperlipidic groups, the MET-HL group presented a significant reduction in retroperitoneal fat compared with the SAL-HL group, although values remained elevated relative to SAL-NL animals. In contrast, periepididymal fat weights were higher in MET-HL compared to MET-NL animals. No statistically significant differences were detected among groups for mesenteric fat. Finally, the MET-NL group did not differ significantly from the SAL-NL group across any of the fat depots analyzed.

### 3.3. Evaluation of Fasting Blood Glucose and Glucose Homeostasis (ITT and ivGTT)

**Figure 5:**
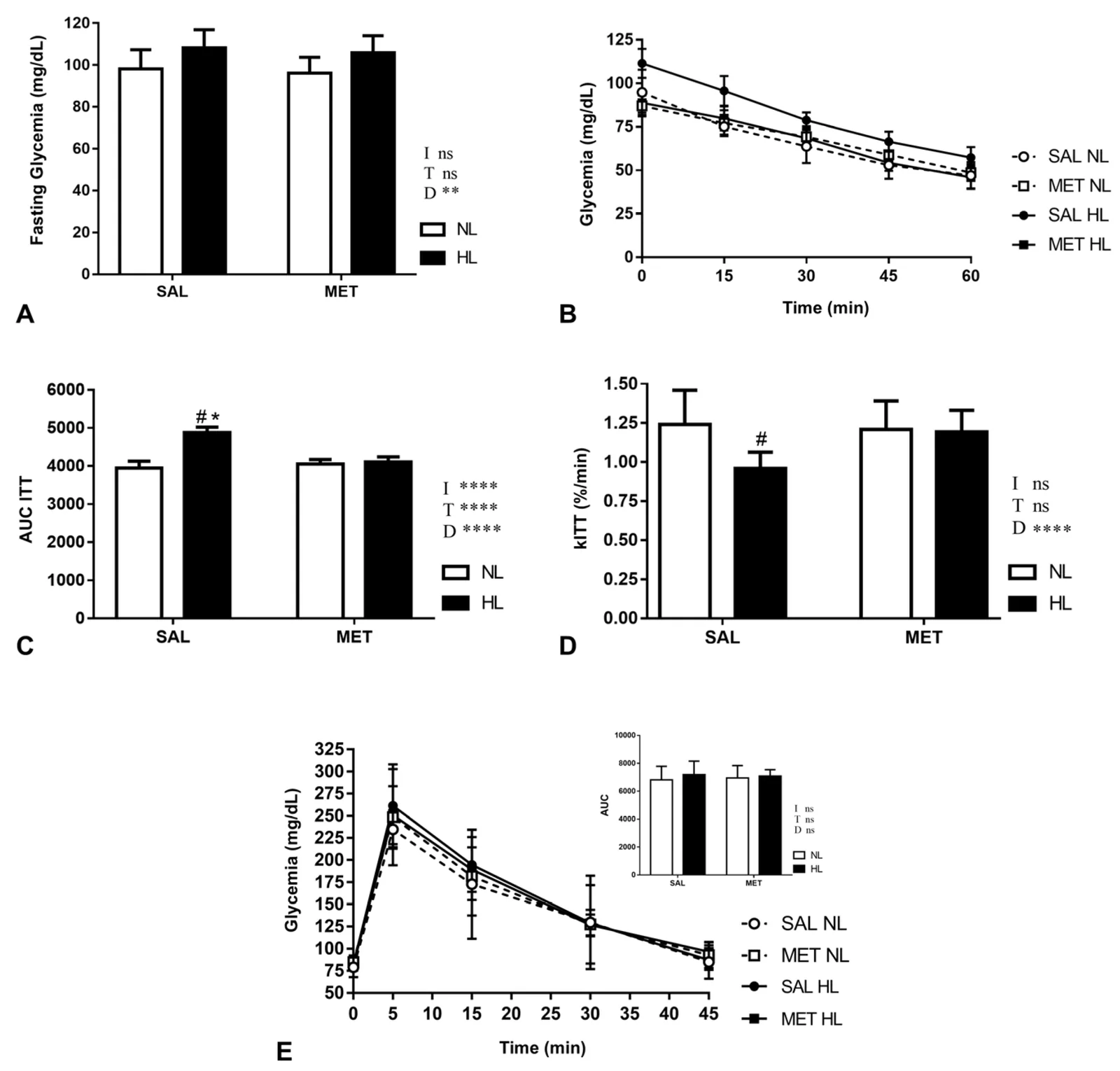
Fasting blood glucose and glucose homeostasis in the experimental groups. (**A**) Fasting blood glucose. (**B**) Area under the curve (AUC) of the intraperitoneal insulin tolerance test (ITT). (**C**) Blood glucose decay rate constant (kITT) derived from the ITT. (**D**) Intravenous glucose tolerance test (ivGTT). (**E**) AUC of the ivGTT. Data are expressed as mean ± SEM (*n* = 20/group). Statistical analysis was performed using two-way ANOVA followed by Bonferroni’s post hoc test. # indicates a significant difference compared with the corresponding NL group; * indicates a significant difference compared with the corresponding SAL group within the same dietary condition; #* indicates significant differences compared with both the corresponding NL group and the corresponding SAL group within the same dietary condition. I, treatment × diet interaction; T, treatment effect; D, diet effect; ns, not significant. # * indicates *p* < 0.05; ** indicates *p* < 0.01; **** indicates *p* < 0.0001.

Animals fed with a HL exhibited higher fasting blood glucose levels compared with their respective NL groups; however, these differences did not reach statistical significance (Figure 5A). In the intraperitoneal insulin tolerance test (ITT), analysis of the area under the curve (AUC) revealed that the SAL-HL group displayed marked insulin resistance, with significantly higher values than both the SAL-NL group and the other experimental groups (Figure 5B). Consistently, the calculation of the kITT confirmed this finding, showing significantly lower values in the SAL-HL group compared with the NL groups (Figure 5C). Metformin treatment appeared to attenuate these alterations under hyperlipidic conditions. The MET-HL group demonstrated a smaller AUC and a higher kITT relative to the SAL-HL group, although these values did not differ significantly from those of the MET-NL group. Moreover, no significant differences were observed between the SAL-NL and MET-NL groups, indicating that metformin alone did not alter insulin sensitivity under normolipidic diet conditions. In the intravenous glucose tolerance test (ivGTT), no statistically significant differences were detected among groups (Figure 5D). Nevertheless, HL-fed animals exhibited numerically higher glucose values compared with NL controls, although without statistical significance. Analysis of the AUC of the ivGTT curves further supported this observation (Figure 5E), with HL groups exhibiting numerically higher AUC values than NL animals, although without statistical significance.

## 4. Discussion

Hyperlipidic diet consumption is a well-established experimental model for the induction of obesity and associated metabolic disturbances [29]. Adult animals exposed to this dietary regimen typically exhibit significant increases in visceral adiposity, particularly in retroperitoneal and epididymal fat depots, along with disturbances in glucose homeostasis and systemic insulin resistance [30]. The lower food intake observed in HL-fed animals should be interpreted considering the higher energy density of the hyperlipidic diet; therefore, reduced food consumption does not necessarily indicate lower caloric intake. These alterations can be demonstrated both indirectly, through insulin tolerance testing, and directly, by glucose tolerance assays, confirming the metabolic impairment induced by chronic lipid overload [30]. In our study, neonatal metformin administration was associated with attenuation of some hyperlipidic diet–related phenotypic alterations. Treated animals exhibited lower body mass gain and reduced retroperitoneal fat accumulation compared to SAL-HL animals. Although these findings do not demonstrate definitive metabolic programming, they support the hypothesis that transient neonatal metformin exposure may modify adult susceptibility to a later obesogenic challenge [31].

Our findings should be interpreted within the framework of developmental plasticity proposed by the DOHaD concept [32]. Rather than producing overt metabolic alterations under physiological conditions, neonatal metformin exposure appeared to modify the response to a subsequent nutritional challenge. This context-dependent effect is consistent with the concept that early-life exposures may alter susceptibility to metabolic dysfunction without necessarily producing an immediate phenotype. Such adaptive responses have been described for several environmental exposures acting during critical developmental windows and are considered central to the DOHaD hypothesis [32].

Metformin has been shown to regulate glucose and lipid metabolism through coordinated effects on mitochondrial metabolism, AMPK activation, suppression of hepatic glucose production, and modulation of GLUT4 trafficking [33]. These pathways may partially explain the reduction in retroperitoneal fat depot mass observed in metformin-treated animals, particularly considering the metabolic characteristics of this adipose depot. However, neither GLUT4 expression nor AMPK activation was evaluated in the present study. Therefore, mechanistic interpretations should be regarded as biologically plausible hypotheses rather than demonstrated pathways, requiring direct experimental confirmation.

The hyperlipidic diet effectively induced systemic insulin resistance, as evidenced by alterations in insulin sensitivity parameters derived from the ITT [34]. Neonatal metformin exposure attenuated these alterations, resulting in higher kITT values in animals challenged with the hyperlipidic diet. Similar improvements in insulin responsiveness have been attributed to coordinated actions of metformin on hepatic glucose production and peripheral glucose utilization [35,36]. Metformin suppresses hepatic gluconeogenesis through mitochondrial mechanisms while enhancing glucose uptake in peripheral tissues via AMPK-dependent pathways [35,36]. Although these mechanisms were not directly evaluated in the present study, they represent plausible biological explanations for the physiological responses observed in metformin-treated animals.

Interestingly, the apparent attenuation of insulin resistance in metformin-treated animals was not accompanied by significant changes in glucose tolerance during the ivGTT. This apparent dissociation may reflect the distinct physiological processes assessed by these tests. Whereas the ITT predominantly evaluates peripheral responsiveness to exogenous insulin, glucose tolerance represents an integrated response involving insulin secretion, peripheral glucose disposal, and regulation of endogenous glucose production. Thus, improved insulin responsiveness may not necessarily translate into detectable differences in overall glucose tolerance. However, circulating insulin concentrations, pancreatic β-cell function, and tissue-specific insulin signaling were not evaluated in the present study; therefore, the mechanisms underlying this differential response cannot be determined from the current data and warrant further investigation. Notably, neonatal metformin administration did not produce detectable metabolic alterations in animals maintained under normolipidic conditions, suggesting that its long-term effects may become more evident in the presence of a subsequent metabolic challenge. This context-dependent response is consistent with the involvement of metformin in cellular energy-sensing pathways, including AMPK-related mechanisms that become particularly relevant under conditions of metabolic stress [37,38].

Although the findings are encouraging, they should not be interpreted as evidence supporting the safety or clinical application of neonatal metformin administration. The neonatal period represents a critical developmental window characterized by rapid growth, tissue differentiation, and maturation of metabolic and neuroendocrine regulatory systems. Pharmacological exposure during this period may therefore have consequences extending beyond the metabolic outcomes evaluated in the present study. Potential effects on organ development, reproductive function, neuroendocrine regulation, and epigenetic programming were not assessed and cannot be excluded. Furthermore, the 100 mg/kg/day dose used in this rat model should not be directly extrapolated to human neonatal dosing because interspecies differences in pharmacokinetics, metabolism, developmental stage, and route of administration preclude a simple dose-to-dose translation. Consequently, the absence of overt abnormalities in the metabolic parameters evaluated here should not be interpreted as evidence of developmental safety. Dedicated studies incorporating both sexes, longer follow-up periods, and comprehensive developmental and molecular assessments will be required to establish the long-term consequences of neonatal metformin exposure. Accordingly, the present findings should not be interpreted as supporting neonatal metformin administration as a preventive or therapeutic strategy in humans [22].

Several limitations should also be considered. First, circulating insulin concentrations, lipid profiles, and adipokines were not assessed, limiting a more comprehensive characterization of the metabolic phenotype and the mechanisms potentially underlying the observed responses. Second, molecular pathways potentially involved in the observed phenotype, including AMPK signaling, GLUT4 regulation, mitochondrial adaptations, and epigenetic modifications, were not investigated. Third, only male offspring were evaluated, preventing assessment of potential sex-specific responses and limiting the generalizability of the present findings to female offspring. Therefore, the metabolic responses observed in this study should be interpreted as specific to male rats, and future studies including both sexes will be necessary to determine whether neonatal metformin exposure produces sex-dependent long-term metabolic effects. Fourth, metabolic outcomes were assessed only during young adulthood, precluding conclusions regarding persistence of these effects throughout aging. In addition, although the individual offspring was considered the experimental unit, litter was not included as an independent factor in the statistical analyses. Therefore, potential clustering of offspring originating from the same litter cannot be excluded and should be considered when interpreting the findings. Future studies should incorporate litter-balanced experimental designs and/or statistical approaches accounting for litter effects. Additionally, although longitudinal body-weight measurements were summarized as individual-animal AUC values, this approach does not formally assess differences in body-weight trajectories or time × treatment interactions. Future studies should therefore incorporate appropriate repeated-measures or mixed-effects analyses to evaluate longitudinal changes in body weight. Finally, the present study was designed as an exploratory investigation and therefore provides hypothesis-generating rather than definitive evidence of developmental metabolic programming. Future studies incorporating broader metabolic, molecular, and developmental assessments will be necessary to provide a more complete characterization of the long-term consequences of neonatal metformin exposure.

Our findings provide preliminary evidence that transient neonatal metformin exposure may modify adult metabolic responses to a subsequent hyperlipidic diet challenge. Future studies integrating molecular analyses, epigenetic profiling, and long-term phenotypic evaluation will be necessary to determine whether neonatal metformin exposure induces persistent developmental adaptations, to clarify the underlying biological mechanisms, and to establish the biological and translational relevance of these findings.

## 5. Conclusions

Our findings provide preliminary evidence that neonatal intraperitoneal metformin administration may modify adult metabolic responses to a subsequent hyperlipidic diet challenge in Wistar rats. Early-life metformin exposure was associated with differences in body weight gain, regional adipose tissue accumulation, and insulin tolerance under hyperlipidic conditions, whereas no substantial metabolic effects were observed under normolipidic conditions. Although the present study was not designed to investigate the molecular mechanisms underlying these effects, the findings support the hypothesis that transient pharmacological exposure during a critical developmental window may influence later susceptibility to diet-induced metabolic alterations. These observations should be interpreted within the exploratory nature of the study, and further investigations are required to elucidate the underlying mechanisms, evaluate the long-term safety of neonatal metformin exposure, and determine the reproducibility and translational relevance of these findings.

## Figures and Tables

**Table 1:** Components of the normolipidic and hyperlipidic diets.

Ingredients (g/kg of Diet)	Normolipidic Diet	Hyperlipidic Diet
Casein	200	200
Sucrose	100	100
Corn starch	427.5	115.5
Dextrinized starch	132	132
Lard	–	312
Soybean oil	40	40
Cellulose 50 50	50	50
Mineral mix (AIN-93)	35	35
Vitamin mix (AIN-93)	10	10
L-cystine	3	3
Choline bitartrate	2.5	2.5

## Data Availability

Data is contained within the article or Supplementary Material.

## Supplementary Materials

The following supporting information can be downloaded at: https://www.mdpi.com/article/10.3390/life16091398/s1. ARRIVAL Guidelines.
